# Effects of dural puncture epidural technique with different drug delivery methods for labor analgesia: A randomized controlled clinical trial

**DOI:** 10.1097/MD.0000000000035217

**Published:** 2023-09-22

**Authors:** Xin Wang, Yaqiu Guo, Meijian Wang

**Affiliations:** a Department of Anesthesiology, The Fifth People’s Hospital of Jinan, Jinan, Shandong, China; b Department of Anesthesiology, Jinan Maternity and Child Care Hospital, Shandong, China.

**Keywords:** clinical trial, drug delivery, dural puncture epidural, labor analgesia

## Abstract

This study aimed to investigate the effect of dural puncture epidural (DPE) combined with small-dose lidocaine for labor analgesia. Parturients were randomly divided into epidural anesthesia (EA), DPE1, and DPE2 groups. In the EA group, 5 mL of 1% lidocaine was administered via conventional L2–L3 puncture catheterization; in the DPE1 group, epidural drug was administered after catheterization using the DPE technique; in the DPE2 group, epidural puncture drug was administered through the epidural puncture needle before catheterization using the DPE technique. The primary outcome was the onset time of analgesia. The secondary outcomes included the numerical rating scale (NRS) scores during uterine contraction before bolus injection of experimental dose (T0) and the second time (T1), the fifth time (T2) and the tenth time (T3) after bolus injection of experimental dose; NRS scores at the second stage of labor (T4) and during perineal suture (T5); operation time of anesthesia; puncture related complications; anesthesia related complications; delivery outcome; use of local anesthesia during vaginal suture; and Apgar score of the neonates. There were 115 women included. The onset time in the DPE2 group was markedly shorter than in the EA and DPE1 groups (*P* < .001). The NRS scores in the DEP2 group at T1 and T4 were significantly lower than in the EA and DEP1 groups (*P* < .001). The overall incidence of puncture related complications in the DEP1 and DEP2 groups was markedly higher than in the EA group (*P* < .05). In dural puncture epidural analgesia, when the experimental dose was injected directly through the epidural puncture needle, the onset time was shorter and the analgesic effect was better as compared to the injection of test dose after inserting the epidural catheter.

## 1. Introduction

Neuraxial analgesia is the first choice for labor analgesia because of its definite analgesic effect and high maternal and child safety. Neuraxial labor analgesia can not only effectively relieve labor pain but also provide quick and favorable anesthetic effect for instrumental delivery or conversion to cesarean section during labor. The techniques used for neuraxial labor analgesia include epidural analgesia (EA), combined spinal–epidural anesthesia (CSEA), and single-shot spinal anesthesia. Although CSEA for labor has shorter onset time and is more effective than simple EA,^[1]^ CSEA can cause maternal hemodynamic instability, fetal bradycardia,^[2,3]^ and many other complications, and thus the epidural technique has been used for labor analgesia in our hospital.

Dural puncture epidural (DPE) is a special technique in which a spinal needle is used to puncture the dura mater and then withdrawn; but no drug is delivered into the subarachnoid space.^[4]^ By delivering the drug into the epidural space through an epidural catheter, the drug can diffuse from the dural hole to the subarachnoid space, thus achieving anesthetic effect. As a new technique, dural puncture epidural anesthesia (DPEA) is used for labor analgesia. In recent years, several clinical studies have investigated labor analgesia. Nonetheless, the role of DPE in labor analgesia remains unclear,^[5]^ and the results of some clinical studies are conflicting. There is evidence showing that DPEA has shorter onset time than EA in labor analgesia and achieves better anesthetic effect.^[6–11]^ However, some studies have revealed that the onset time in DPEA is not shortened,^[9,12,13]^ but the incidence of complications increases.^[9]^ In the present randomized controlled clinical trial, the effect of DPEA with the same model of dural puncture needle (25 G) and 2 different ways of drug delivery was compared aiming to elucidate the onset of action and safety of DPEA for labor analgesia.

## 2. Material and methods

### 2.1. Study population

This was a prospective, randomized, double blind clinical trial which was approved by the institutional Ethics Committee of the fifth People Hospital of Jinan (Ethics No: 21-5-01). (11/12/2021) and Jinan Maternal and Children Care Hospital, Shandong first Medical University (Ethics No: 2021-1-041). (11/15/2021). The written informed consent was obtained from all subjects before the study. The trial was registered at the Chinese Clinical Trials Registry (ChiCTR2100053337; XinWang, Yaqiu Guo; 11/19/2021).

In this study, 120 healthy parturients who were scheduled to undergo labor analgesia in the fifth People Hospital of Jinan (n = 60) and Jinan Maternity and Children Care Hospital (n = 60) from November 2021 to February 2022 were enrolled. The inclusion criteria were as follows: patients had American Society of Anesthesiologists grade I–II and New York Heart Association Heart Function grade I; the height was 155 to 175 cm; the weight was 55 to 95 kg; subjects had a singleton pregnancy of 37 to 42 weeks; subjects voluntarily received epidural labor analgesia when the cervical diameter was 1 to 3 cm; subjects had no cardiovascular diseases, endocrine diseases, mental diseases, and deformities. The exclusion criteria were as follows: subjects had induced labor; the body mass index was >40 kg/m^2^; there were contraindications to EA or subarachnoid anesthesia; the pain visual analog score numerical rating scores (NRS) was ≤3 when requiring labor analgesia; anesthesia was ineffective. Participants were also excluded in the event of epidural catheter into subarachnoid space, or an inadvertent dural puncture using the epidural needle.

A total of 120 parturients were randomly divided into EA group (n = 40), DPE1 group (n = 40), and DPE2 group (n = 40) according to the computer-generated random numbers. The group assignment was hidden in an opaque, numbered envelope and opened by the anesthesiologist before the start of analgesia, and the investigators who evaluated the results were blind to the grouping. Two full-time anesthesiologists performed the puncture operation, and both were anesthesiologists with an intermediate professional title and had experience in anesthesia for more than 6 years. The intervertebral space of lumbar vertebrae 2 to 3 (L2–L3) was selected for puncture of the epidural space with a 16-G puncture needle. After using a loss of resistance to saline technique to confirm the success of epidural puncture, in the EA group, the experimental dose was delivered by bolus injection after the epidural catheter was inserted into the epidural space in the cranial direction by 4 cm; the experimental dose was 5 mL of 1% lidocaine, and the bolus injection was performed at 1 mL/second. In the DPE1 group, the dura mater was punctured with a 25-G dural puncture needle to access the subarachnoid space, and the successful puncture was determined as cerebrospinal fluid outflow. Then, the puncture needle was withdrawn, the epidural catheter was inserted into the epidural space by 4 cm in the cranial direction, and the experimental dose was delivered by epidural catheter. The experimental dose here was the same as that in the EA group. The injection was performed at 1 mL/second. In the DPE2 group, the dura mater was punctured with a 25-G dural puncture needle to reach the subarachnoid space, and the puncture needle was removed. The experimental dose was directly delivered by bolus injection from the epidural puncture needle, and the experimental dose was the same as that in the EA and DPE1 groups. The injection was also performed at 1 mL/second. After drug injection, the epidural catheter was inserted into the epidural space by 4 cm in the cranial direction. After bolus injection, if no abnormality was found within 10 minutes, programmed intermittent epidural bolus plus patient-controlled EA was initiated. According to the height of the parturient, the background dose was set as 0.083% ropivacaine at 10 mL/50 minutes, the patient-controlled dose was 8 mL, and the locking time was 30 minutes.

### 2.2. Primary outcome

The onset time of analgesia in each parturient was observed and recorded. The onset time was the interval from the end of bolus injection to significant relief of labor pain (NRS score ≤3).

### 2.3. Secondary outcomes

The NRS scores of the parturient during uterine contraction before bolus injection of the experimental dose (T0) and the second time (T1), the fifth time (T2), and the tenth time (T3) after bolus injection of experimental dose; the NRS scores at the second stage of labor (T4) and during perineal suture (T5) were recorded. The anesthesia operation time of each parturient was also recorded. The anesthesia operation time was defined as the time from putting on sterile gloves to taking off sterile gloves after puncture. The puncture complications, anesthesia complications, delivery outcome, use of local anesthesia during vaginal suture, and Apgar score of the neonate were recorded for each parturient. The puncture complications included paresthesia, displacement of epidural catheter into the subarachnoid space, and displacement of epidural catheter into the blood vessel. The anesthesia complications included fever, hypotension, nausea and vomiting, lower limb limitation of motion, lower limb paresthesia, postoperative headache, and decreased fetal heart rate. Fever was defined as an increase in maternal body temperature (axillary body temperature >37.3°C) after anesthesia. Limitation of motion in the lower limbs was defined as a Bromage scale score ≥1. Hypotension was defined as a decrease in systolic blood pressure by ≥15% of baseline blood pressure.

### 2.4. Statistical analysis

This was a double-center, double-blind, randomized, controlled clinical trial. According to previously reported,^[7]^ the average onset time of the drug in 3 groups was approximately 6 minutes, 10 minutes, and 10 minutes, respectively, and the standard deviation was 2 in 3 groups. The sample size was calculated using Power Analysis & Sample Size 10.01 software (SPSS Inc., Chicago, IL) with a significance level α = 0.05, power (1-β) = 0.9, allocation ratio of 1:1:1, and a drop-off rate of 20%. Results showed at least 22 patients were required in each group to detect a statistically significant difference between each pair of groups (a total of 81 patients were required in 3 groups). There were 40 patients in each group, and thus 120 patients were recruited into present study.

Statistical analysis was performed using the Statistical Package for the Social Sciences version 25.0 (IBM Corp., Armonk, NY). Categorical data are described as the frequency or percentage, and were compared with Chi-squared test or fisher exact test. The mean ± standard deviation was used for the description of quantitative data with normal distribution. Furthermore, 1-way analysis of variance was used comparisons among groups, and Tukey method was used for the comparison between groups. For quantitative data without normal distribution or with heterogeneity of variance, comparisons were performed using the Kruskal–Wallis H rank-sum test, and pairwise comparisons were performed using the Nemenyi method. A value of *P* < .05 was considered statistically significant.

## 3. Results

**Figure 1.**
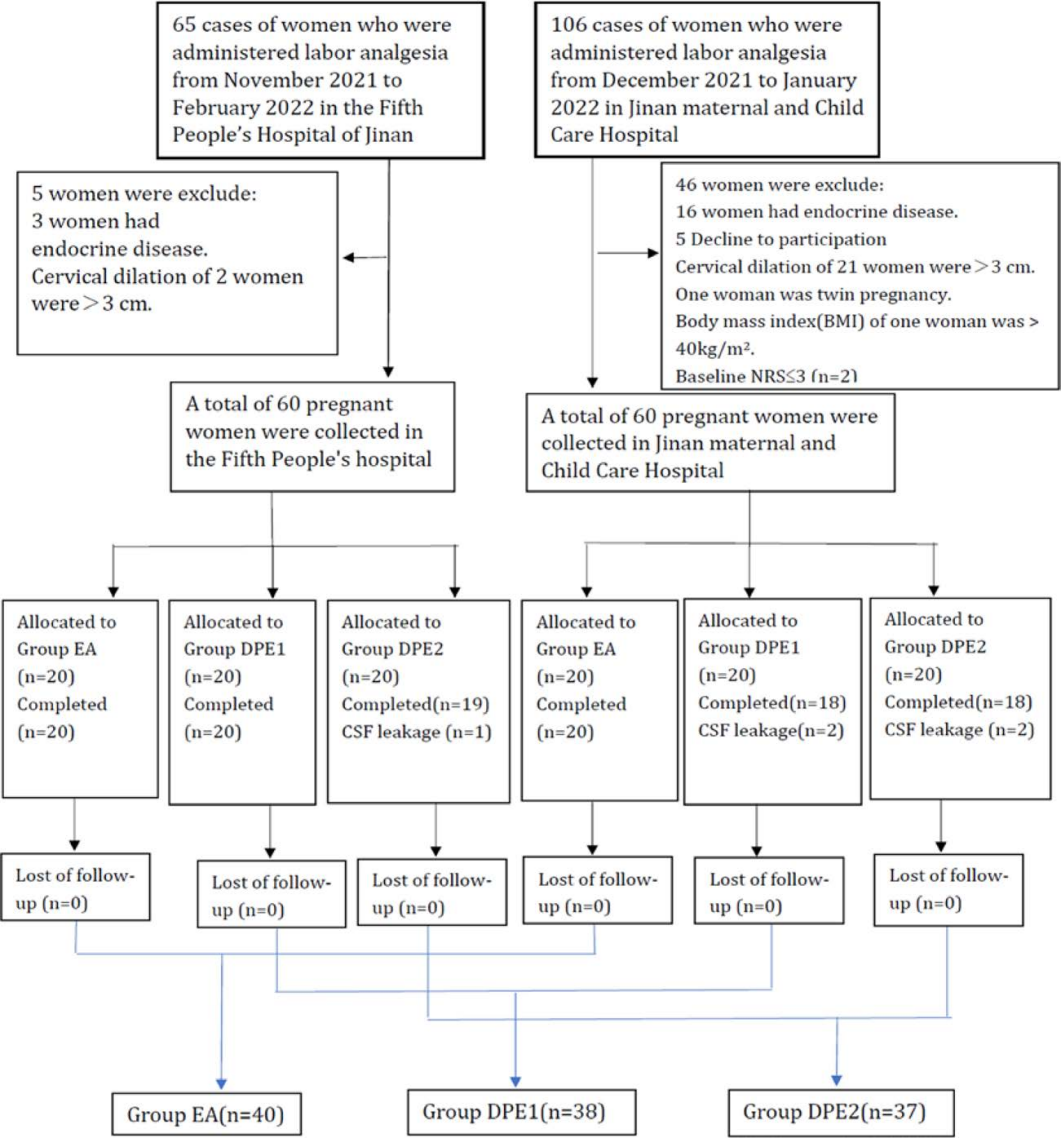
CONSORT trial flow diagram.

From November 2021 to March 2022, 171 parturients were screened, and 51 were excluded from these parturients (Fig. 1). A total of 120 parturients were included in this study, including 60 patients from the fifth People Hospital of Jinan and 60 patients from Jinan Maternity and Children Care Hospital. These parturients were randomly divided into 3 groups: EA, DPE1, and DPE2 groups. 5 parturients were excluded after randomization (Fig. 1). Data were thus collected from 115 subjects. The age, height, weight, body mass index, gestational age, and cervical diameter were comparable among 3 groups (*P* > .05) (Table 1).

**Table 1:** Demographic and baseline characteristics.

	EA (n = 40)	DPE1 (n = 40)	DPE2 (n = 40)	*F/χ* ^2^	*P* value
Age (yr)	26.45 (3.60)	25.90 (3.28)	26.58 (3.62)	0.420	.658
Height (m)	163.18 (4.48)	162.15 (4.70)	162.73 (4.70)	0.517	.598
Weight (kg)	74.13 (10.20)	73.99 (6.95)	73.69 (9.30)	0.026	.974
Body mass index (kg/m^2^)	27.85 (3.02)	28.15 (2.29)	27.82 (3.21)	0.164	.849
Gestational time (wk)	39.84 (1.11)	39.81 (1.07)	40.10 (0.90)	0.977	.379
Cervical dilation at the time of neuraxial placement (cm)	2.25 (0.67)	2.05 (0.60)	2.10 (0.59)	1.127	.328

Values are expressed as mean (SD).

DPE = dural puncture epidural, EA = epidural anesthesia, SD = standard deviation.

### 3.1. Primary outcomes

There was significant difference in the onset time among 3 groups (*P* < .05), with the onset time in the DPE2 group being significantly shorter than in the EA and DPE1 groups; there was no difference between EA group and DPE1 group (Table 2).

**Table 2:** Primary outcome.

	EA (n = 40)	DPE1 (n = 40)	DPE2 (n = 40)	*F/χ* ^2^	*P* value
The onset time (min)	9.68 (0.83) ^b^	9.90 (0.93) ^*b*^	2.80 (0.46) ^*a*^	84.724	<.001

The superscripts in different letters indicate that the difference was statistically significant, with the mean value *b > a*. The Kruskal–Wallis H rank-sum test was used for intergroup comparison of onset time, and the Nemenyi method was used for pairwise comparison.

DPE = dural puncture epidural, EA = epidural anesthesia.

### 3.2. Secondary outcomes

There was no significant difference (*P* > .05) in the NRS score at T0, T2, and T3 among 3 groups, but significant difference (*P* < .05) in the NRS score was noted at T1 and T4 among 3 groups. Moreover, the NRS score in the DPE2 group at T1 and T4 was significantly lower than in the EA and DPE1 groups. There was significant difference (*P* < .05) in the anesthesia operation time among 3 groups: the anesthesia operation time in the DPE2 group was significantly longer than in the EA and DPE1 groups; it in the DPE1 group was significantly longer than in the EA group. There was no significant difference in the Apgar score at 1 minute and 5 minutes among 3 groups (*P* > .05). There was also no marked difference in the delivery outcome among 3 groups (*P* > .05). Additionally, the fetal position also comparable among 3 groups (*P* > .05) (Table 3).

**Table 3:** Secondary outcome.

	EA (n = 40)	DPE1 (n = 40)	DPE2 (n = 40)	*F/χ* ^2^	*P* value
NRS (T0), mean (SD)	5.78 (0.73)	5.75 (0.71)	5.95 (0.75)	0.891	.413
NRS (T1), mean (SD)	5.83 (0.71)	5.80 (0.69)	2.90 (0.30)	316.785	<.001
NRS (T2), mean (SD)	0.88 (0.46) ^*a*^	0.85 (0.53) ^*a*^	1.08 (0.47) ^*b*^	2.519	.085
NRS (T3), median (Q1,Q3)	0.00 (0.00,0.00)	0.00 (0.00,0.00)	0.00 (0.00,0.00)	0.642	.726
NRS (T4), mean (SD)	4.15 (0.36) ^*b*^	4.13 (0.40) ^*b*^	3.73 (0.55) ^*a*^	11.351	<.001
Abnormal fetal position, n (%)	6 (15.00)	5 (12.50)	6 (15.00)	0.137	.934
Cesarean delivery, n (%)	6 (15.00)	5 (12.50)	4 (10.00)	0.137	.796
Apgar (1 min), mean (SD)	9.88 (0.33)	9.85 (0.36)	9.83 (0.45)	0.170	.844
Apgar (5 min), mean (SD)	10 (–)	10 (–)	10 (–)	–	–
Puncture time (min), mean (SD)	9.03 (1.72)	11.30 (0.88)	12.33 (1.37)	61.200	<.001

The superscripts in different letters indicate that the difference was statistically significant, with the mean value *b > a*.

DPE = dural puncture epidural, EA = epidural anesthesia, NRS = numerical rating scores, SD = standard deviation.

There were no significant differences in the puncture complications, including paresthesia, displacement of epidural catheter into the subarachnoid space, and displacement of epidural catheter into the blood vessels (*P* > .05), among 3 groups. However, significant difference was noted in the total number of puncture complications (*P* < .05): the overall incidence of puncture complications in the DPE1 and DPE2 groups was significantly higher than in the EA group. There was significant difference in the incidence of lower limb paresthesia after anesthesia among 3 groups (*P* < .05): the incidence of lower limb paresthesia in the DPE2 group was significantly higher than in the EA and DPE1 groups. There was no marked difference in the anesthesia complications among 3 groups (*P* > .05). Maternal fever, hypotension, nausea and vomiting, lower limb limitation of motion, postoperative headache, and fetal bradycardia were not observed in 3 groups. There was marked difference in the utilization of local anesthesia for vaginal suture among 3 groups (*P* < .05): significantly less subjects received local anesthesia for vaginal suture in the DPE2 group than in the EA and DPE1 groups (Table 4).

**Table 4:** Incidence of AE (n [%]).

	EA (n = 40)	DPE1 (n = 40)	DPE2 (n = 40)	*P* value
Puncture complications				
Paresthesia	0 (0.00)	4 (10.00)	5 (12.50)	.075
Epidural catheter into subarachnoid space	0 (0.00)	2 (5.00)	3 (7.50)	.368
Epidural catheter into the blood vessels	1 (2.50)	2 (5.00)	2 (5.00)	.999
The total of puncture complications	1 (2.50)	8 (20.00)	9 (22.50)	.035
Anesthesia complications				
Maternal fever	3 (7.50)	4 (10.00)	4 (10.00)	.999
Lower limb limitation of motion	0 (0.00)	0 (0.00)	0 (0.00)	–
Hypotension	0 (0.00)	0 (0.00)	0 (0.00)	–
Nausea and vomiting	0 (0.00)	0 (0.00)	0 (0.00)	–
Postoperative headache	0 (0.00)	0 (0.00)	0 (0.00)	–
Lower limb paresthesia	0 (0.00)	0 (0.00)	4 (10.00)	.033
Fetal bradycardia	2 (5.00)	1 (2.50)	3 (7.50)	.870
The total of anesthesia complications	5 (12.50)	5 (12.50)	11 (27.50)	.170
Utilization rate of local anesthesia for vaginal suture	40 (100.00)	40 (100.00)	27 (67.50)	<.001

DPE = dural puncture epidural, EA = epidural anesthesia.

## 4. Discussion

Our study showed that in DPEA, the onset time of anesthesia was shorter when the experimental dose was injected directly through the epidural puncture needle and the analgesic effect was better when the test dose was injected after inserting the epidural catheter. In DPEA, when the drug was injected after catheterization, there was no significant difference between DPEA and EA with respect to the analgesic effect.

The onset time of analgesia in the DPE2 group was shorter than in the EA group, and the NRS score at T1 and T4 was significantly lower than in the EA group. This indicates that the drug diffused into the subarachnoid space when the experimental dose was injected through the epidural puncture needle, which was responsible for the subarachnoid anesthesia. There was no significant difference in the analgesic effect between DPE1 and EA groups. This indicates that the drug did not diffuse into the subarachnoid space, or the amount of diffused drug was significantly smaller when the drug was injected after the epidural catheter was placed, and therefore the effect of subarachnoid anesthesia was minimal. This might be related to the position and direction of drug injection. In this study, the position of drug delivery in the DPE2 group was anterior to the dural hole, whereas that in the DPE1 group was anterosuperior to the dural hole. Thus, the drug delivery in the DPE2 group was closer to the dural hole than in the DPE1 group. Hence, it could easily spread to the subarachnoid space. When the epidural catheter was used for drug delivery, the drug diffused from multiple directions because the catheter had 3-sided holes. When the drug was injected directly from the epidural puncture needle, the entire drug volume diffused anterior to the dural hole. Hence, the probability of drug diffusion into the subarachnoid space in the DPE2 group was higher than in the DPE1 group.

Gupta et al^[12]^ found that the analgesic effect of DPEA was not improved as compared to continuous EA, and the incidence of paresthesia during EA was higher. These were consistent with our findings. However, the study of Gupta et al aimed to compare DPEA and EA with programmed intermittent epidural bolus. The analgesic effect of DPEA with the drug injected after catheterization was similar to that of EA. Compared with EA alone, DPEA technique had a significantly higher incidence of puncture complications and longer operation time. Although the analgesic effect in the DPEA2 group was better than in the EA group at T1 and T4, the incidence of lower limb paresthesia increased significantly during anesthesia.

Contreras et al^[14]^ found that the onset time of DPEA using 25-G dural puncture needle was shorter than that with a 27-G dural puncture needle. In this study, the same combined spinal–epidural puncture kit was used in 120 subjects, the 16 G needle was used for epidural puncture, the 25 G needle was used for dural puncture, and the puncture was performed at interspace L2–L3. Two anesthetists had experience in anesthesia for more than 6 years, and another 2 midwives responsible for the data collection had experience for more than 10 years. Two study centers cooperated to avoid bias caused by objective and subjective factors as much as possible.

A study of Chau et al^[9]^ showed, when CSEA, DPEA, and EA were used for labor analgesia, CSEA had the shortest onset time, there was no significant difference in the onset time between DPE and EA, and DPE had better blocking effect than EA. Their results were different from ours, which might be related to the dose of anesthetic. In their study, the initial dosing consisted of 20 mL of 0.125% bupivacaine and 2 μg/mL of fentanyl administered through the catheter, which was significantly larger than that used in our experiment. Moreover, the dose in their study exceeded the recommended in expert consensus on labor analgesia in China.^[15]^ Cappiello et al^[6]^ found that DPEA with a 25-G spinal needle improved the sacral spread and onset in laboring nulliparous patients. They did not use the test amount. After successful epidural puncture, 12 mL of bupivacaine was directly injected at 2.5 mg/mL through the epidural catheter, and the concentration and volume of the drug were more than twice those used in our experiment.

This study had several limitations. First, the maternal satisfaction was not assessed in the present study. The NRS score showed that parturients in 3 groups achieved sufficient analgesia 30 minutes after anesthesia, but whether the parturients were satisfied with the overall anesthetic effect was still unclear. Second, the time points at which NRS score was obtained were scattered and should be more concentrated. In our study, NRS score was obtained at 5 time points, in which T3 and T4 were at the second stage of labor and the time of vaginal suture, respectively. The time of the first stage of labor was different among parturients. Hence, the time of T3 and T4 from the time of implementation of anesthesia was different, which could lead to deviations in NRS scores at T3 and T4. Third, after anesthesia, the anesthesia level was not confirmed. Asymmetry of bilateral block areas was not found. Fourth, the times of patient-controlled analgesia were not recorded in these parturients. Thus, it is infeasible to assess the difference in the total dose used in 3 groups.

## 5. Conclusion

In summary, our findings indicate there is no difference in the anesthetic effect between DPEA with the drug injected after catheterization and EA during labor analgesia. The analgesic effect of DPEA is better than that of EA when the epidural puncture needle is used to inject the experimental dose of the drug. However, when DPE was used for labor analgesia, the incidence of puncture and anesthetic complications significantly increases.

## Acknowledgments

The authors thank midwife Xiuqin Sun and midwife Yali Zhou for their support and assistance during this study.

## Author contributions

**Data curation:** Xin Wang.

**Formal analysis:** Xin Wang, Yaqiu Guo.

**Investigation:** Xin Wang, Yaqiu Guo.

**Methodology:** Yaqiu Guo.

**Supervision:** Meijian Wang.

**Validation:** Meijian Wang.

**Writing – original draft:** Meijian Wang.
